# Finding meaning in bioimpedance for people treated with hemodialysis

**DOI:** 10.1590/2175-8239-JBN-2023-E010en

**Published:** 2023-09-18

**Authors:** David Keane, Sintra Eyre

**Affiliations:** 1 University of Galway, HRB Clinical Research Facility Galway, CÚRAM SFI Research Centre for Medical Devices, Galway, Ireland.; 2 Sahlgrenska University Hospital, Institute of Medicine, Department of Clinical Nutrition, Gothenburg, Sweden.

Stripped back to basics, bioimpedance is a very simple technology. When an alternating current is applied across biological tissue, the impedance to the current is strongly dependent on the characteristics of the tissue. This allows us to use the measured impedance to obtain information on body composition. However, translating this fundamental principle into clinically meaningful information is less simple. A lack of understanding of the assumptions and uncertainties associated with measured parameters and too little consideration of how to incorporate the results into clinical decision making have meant that, despite its huge potential, bioimpedance is still not widely accepted as an evidence-based diagnostic tool in hemodialysis.

So what does a dialysis service need to understand about bioimpedance? For those interpreting the results, it is probably sufficient to ensure that the test is applied within recommended measurement routines for the device and an appropriate clinical decision pathway that acknowledges measurement uncertainty. However, for those involved in obtaining and developing these pathways, a greater understanding is needed.

Possibly most important is the ability to navigate the number of devices available and the variables that are generated during a measurement. Some devices give “raw” data, such as phase angle (PhA), which come directly from the impedance measurement. Most devices will also report more clinically intuitive parameters, such as extracellular water (ECW), which are calculated from the measured impedance using one or more prediction equations. These empirical equations are based on measurements from a reference population and their validity depends on how well an individual reflects the characteristics of the reference population. It is therefore important for the user to know the characteristics of the reference population and exercise increased caution if it is less representative of the subjects being measured.

It is also important to understand the significance of body composition models that are used to generate clinically meaningful information from basic variables such as ECW. In hemodialysis, bioimpedance is most commonly used for monitoring nutritional status and fluid status. Unfortunately, the 2-compartment model (fat mass (FM) and fat-free mass (FFM), which underpins most bioimpedance devices, cannot distinguish fluid overload from FFM. This makes it challenging to differentiate between muscle wasting and fluid accumulation, both of which are highly prevalent in this population.

In this issue, Zeni et al.^1^ report a prospective, observational study evaluating bioimpedance for nutritional and volume status assessment in a single-center cohort in Brazil. The study clearly demonstrates the issues raised above. First, a broad range of variables is reported, including “raw” bioelectrical data (PhA) and derived parameters such as extra-and intra-cellular water (ECW and ICW) volumes. Concerns over the population used to generate the prediction equations are rightly highlighted, although analogous concerns apply to populations used to generate “normal” values for raw parameters such as PhA. The authors address concerns about normal ranges by suggesting a focus on longitudinal changes in measurements as the best way to obtain clinically relevant information to support clinical care.

The impact of using body composition models that assume euvolemia is neatly demonstrated by measuring changes in fluid status and nutritional parameters between pre- and post-HD. ICW is stable after ultrafiltration, while ECW and total body water (TBW) are reduced and PhA increases, highlighting the dependence of PhA on fluid as well as nutritional status. Analyses stratified by age demonstrate the impact of age-related loss of lean tissue on bioimpedance-derived parameters, such as ECW/TBW. The authors clearly show that ECW/TBW and PhA are associated with mortality, but to be able to intervene, the effect of age, lean tissue mass, malnutrition, and obesity must be untangled.

**Figure 1:**
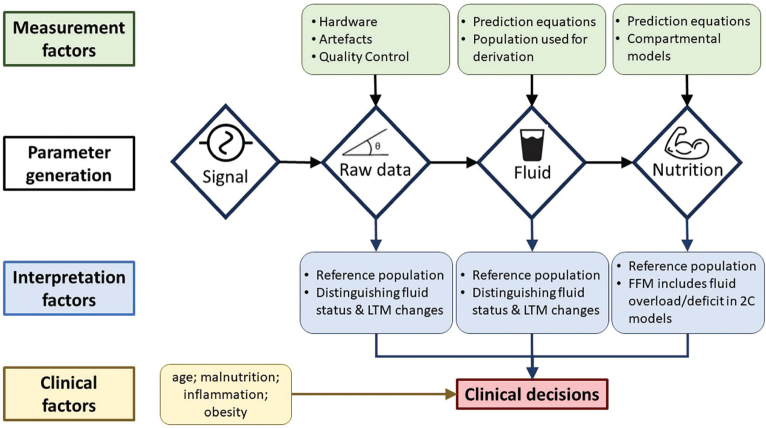
Summary of key factors that impact bioimpedance measurement and interpretation in the clinical setting. LTM: lean tissue mass; FFM: fat free mass; 2C: two compartment.

What are the implications of these observations in practice? Clinically, it seems prudent to integrate the use of bioimpedance with clinical assessment allowing a holistic assessment of fluid and nutritional status, rather than providing a simple target applicable to all patients^2,3^ (Figure 1). Interpreting a decreased PhA or increased ECW/TBW ratio as a result of excess fluid without considering possible nutritional changes could lead to excessive ultrafiltration, residual renal function loss, and other sequela of end-organ hypoperfusion^4^. The use of clinical decision support tools such as the Recova^®^ tool^5^ may facilitate interpretation of bioimpedance in conjunction with important clinical variables, such as age, nutritional status, underweight or overweight, and inflammation.

From a research perspective, bioimpedance has largely struggled to improve hard outcomes in interventional studies^2^, although it is important to note that the way that bioimpedance is used to support clinical decision making varies greatly among studies. It would be wise to carefully consider how to evaluate the use of bioimpedance as part of clinical decision pathways, an application that fits the definition of a complex intervention^6^. Despite the lack of evidence, bioimpedance is being widely used in practice. In certain countries, where the use of bioimpedance is highly prevalent, researchers should consider the extent to which it would be acceptable to clinicians and patients to withdraw the use of the technology in the control arm of an interventional study.

Considering the differences in devices, parameters, models, and applications, there are challenges in generating a coherent evidence base to support the inclusion of bioimpedance in clinical practice guidelines. This should only reinforce the need for understanding the limitations of the technology, standardization of protocols for the hemodialysis population, and reference values relevant to this population to support evidence-based translation into practice^7^.
